# Inflammatory response in dairy cows caused by heat stress and biological mechanisms for maintaining homeostasis

**DOI:** 10.1371/journal.pone.0300719

**Published:** 2024-03-25

**Authors:** Hana Kim, Jang-Hoon Jo, Hong-Gu Lee, Woncheoul Park, Hak-Kyo Lee, Jong-Eun Park, Donghyun Shin

**Affiliations:** 1 Department of Animal Biotechnology, Jeonbuk National University, Jeonju, Jeollabuk-do, Republic of Korea; 2 Department of Animal Science and Technology, Sanghuh College of Life Sciences, Konkuk University, Seoul, Republic of Korea; 3 Division of Animal Genomics and Bioinformatics, National Institute of Animal Science, Rural Development Administration, Wanju, Jeollabuk-do, Republic of Korea; 4 Department of Animal Biotechnology, College of Applied Life Science, Jeju National University, Jeju, Jeju-do, Republic of Korea; 5 Agricultural Convergence Technology, Jeonbuk National University, Jeonju, Jeollabuk-do, Republic of Korea; University of Hawai’i at Manoa, UNITED STATES

## Abstract

Climate change increases global temperatures, which is lethal to both livestock and humans. Heat stress is known as one of the various livestock stresses, and dairy cows react sensitively to high-temperature stress. We aimed to better understand the effects of heat stress on the health of dairy cows and observing biological changes. Individual cows were divided into normal (21–22 °C, 50–60% humidity) and high temperature (31–32 °C, 80–95% humidity), respectively, for 7-days. We performed metabolomic and transcriptome analyses of the blood and gut microbiomes of feces. In the high-temperature group, nine metabolites including linoleic acid and fructose were downregulated, and 154 upregulated and 72 downregulated DEGs (Differentially Expressed Genes) were identified, and eighteen microbes including *Intestinimonas* and *Pseudoflavonifractor* in genus level were significantly different from normal group. Linoleic acid and fructose have confirmed that associated with various stresses, and functional analysis of DEG and microorganisms showing significant differences confirmed that high-temperature stress is related to the inflammatory response, immune system, cellular energy mechanism, and microbial butyrate production. These biological changes were likely to withstand high-temperature stress. Immune and inflammatory responses are known to be induced by heat stress, which has been identified to maintain homeostasis through modulation at metabolome, transcriptome and microbiome levels. In these findings, heat stress condition can trigger alteration of immune system and cellular energy metabolism, which is shown as reduced metabolites, pathway enrichment and differential microbes. As results of this study did not include direct phenotypic data, we believe that additional validation is required in the future. In conclusion, high-temperature stress contributed to the reduction of metabolites, changes in gene expression patterns and composition of gut microbiota, which are thought to support dairy cows in withstanding high-temperature stress via modulating immune-related genes, and cellular energy metabolism to maintain homeostasis.

## Introduction

Temperature is major factor that affects livestock production and are associated with dairy cattle quality. Climatic changes result in abnormal weather conditions that increase temperatures worldwide, causing a reduction in livestock production and leading to economic losses [1]. In recent years, unprecedented climatic changes have frequently occurred with extreme weather, which can adversely affects the health of cattle [2]. Dairy cattle respond sensitively to heat stress, showing changes in product composition, physiology, and behavior [3]. Moreover, high-production and sensitivity to heat stress in dairy cattle indicate that positive correlation, and temperatures between 15–25 °C on livestock farm is possible to harvest maximum production at low cost maintaining normal condition in dairy cattle [4]. The temperature–humidity index (THI) is used to estimate heat stress conditions using temperature and relative humidity by calculating improvement in THI values in cattle [5]. THI 70–74 may be affected by heat stress, THI >75–78 is considered the threshold for normal body temperature and biological mechanisms in dairy cattle [1, 6]. In addition, the temperature between 25–26 °C is considered the threshold in normal conditions, and environments with high humidity and temperatures above the threshold elicit heat physiological and behavioral stress responses, which results in alteration of blood composition, cellular energy metabolism, immune system, oxidative stress, and hormonal level [4, 7]. Generally, exposure to a heat stress environment negatively affects growth performance, including daily weight gain and feed intake in various animals [8]. Recent evidence focusing on environmental temperature reported that animal health can be sacrificed by physiological and behavioral changes at high temperatures, resulting in biological problems, including cellular redox imbalance caused by oxidative stress [9]. Furthermore, maintaining homeostasis from heat stress is important for animal health and economic productivity. In general, livestock animals can tolerate heat stress via their heat stress response [10]. It is modulated by the endocrine system with hormonal secretion and is well indicated in chronic heat stress conditions with alteration of biological pathways and physiologic states; these processes are known to take several weeks [11]. The homeostasis mechanism of livestock animals was developed to minimize negative impacts and is shown as phenotypes by biological states, which are processes that acquire homeostasis under heat stress conditions [12]. Also, the discovery of various biological changes by heat stress can be utilized as biological markers in animal breeding processes [13, 14]. Candidate markers have been partially investigated in terms of their availability in metabolites, transcriptomes, and metagenomes [15–17].

The composition and amount of blood metabolites are influenced by heat stress, and hormones, sugars, amino acids, and fatty acids in blood have been proposed as heat stress indicators [18–21]. In addition, heat stress influences gene expression patterns related to metabolism, immune system, and inflammatory responses, resulting in physiological changes in the composition and amount of blood metabolites [14, 22, 23]. Some metabolites including fructose and linoleic acid provide cellular protection, inflammatory responses, and energy sources, can accelerate metabolic processes under stress conditions [24–27]. Moreover, fructose is reported to activate immune system through production of advanced glycation end-products, and uric acid, which is known to be generated under fructose metabolism process, is involved in activation of immune cells, and plays a role in selective antioxidant against oxidative stress [28, 29]. Metabolites can also play the role of transcription factors and modulate gene expression through binding to the metabolite-sensing mRNA [30–32]. Although it is difficult to modulate all metabolites by gene expression alone, different growth conditions in animals may indicate significant associations between metabolites and mRNA [33]. Recent research has shown that the relationships between blood metabolite and microbiota influence mutual metabolism and provide evidence of an interactive effect between host physiology and microbial composition [34–36]. In addition, the host and microbes have developed beneficial relationships, and intestinal microbes promote the development of the intestinal epithelium and immune system, which balance immunoglobulin A and antimicrobial peptides [37, 38]. In particular, intestinal microbes have been suggested to interact with the host immune system through the gut-brain axis based on the neuro-immuno-endocrine with hypothalamic-pituitary-adrenal (HPA) axis, and are able to boost the immune response for intestinal homeostasis under chronic stress conditions, which is known to be important for the host [39–41]. The HPA axis includes corticotropin-releasing hormones, adrenocorticotropic hormones, and corticosteroids, and their secretion is important for maintaining homeostasis for heat stress tolerance [42, 43]. Hence, these microbes secrete metabolites, neurotransmitters, through the gut-brain axis and may regulate the release of cytokines and chemokines, which is activated by the HPA axis under heat stress conditions [9, 44]. The association between metabolites, genes, and microorganisms has been studied to discover interactive relationships at a wide level, and determining their relationships could possibly contribute to understanding interactive mechanisms at the metabolome, transcriptome, and microbiome levels [45–49]. The objective of this study was to investigate the biological changes induced by interactive relationships among metabolites, differentially expressed genes (DEGs) and abundant microbes under heat stress conditions in dairy cattle.

## Results

**Fig 1:**
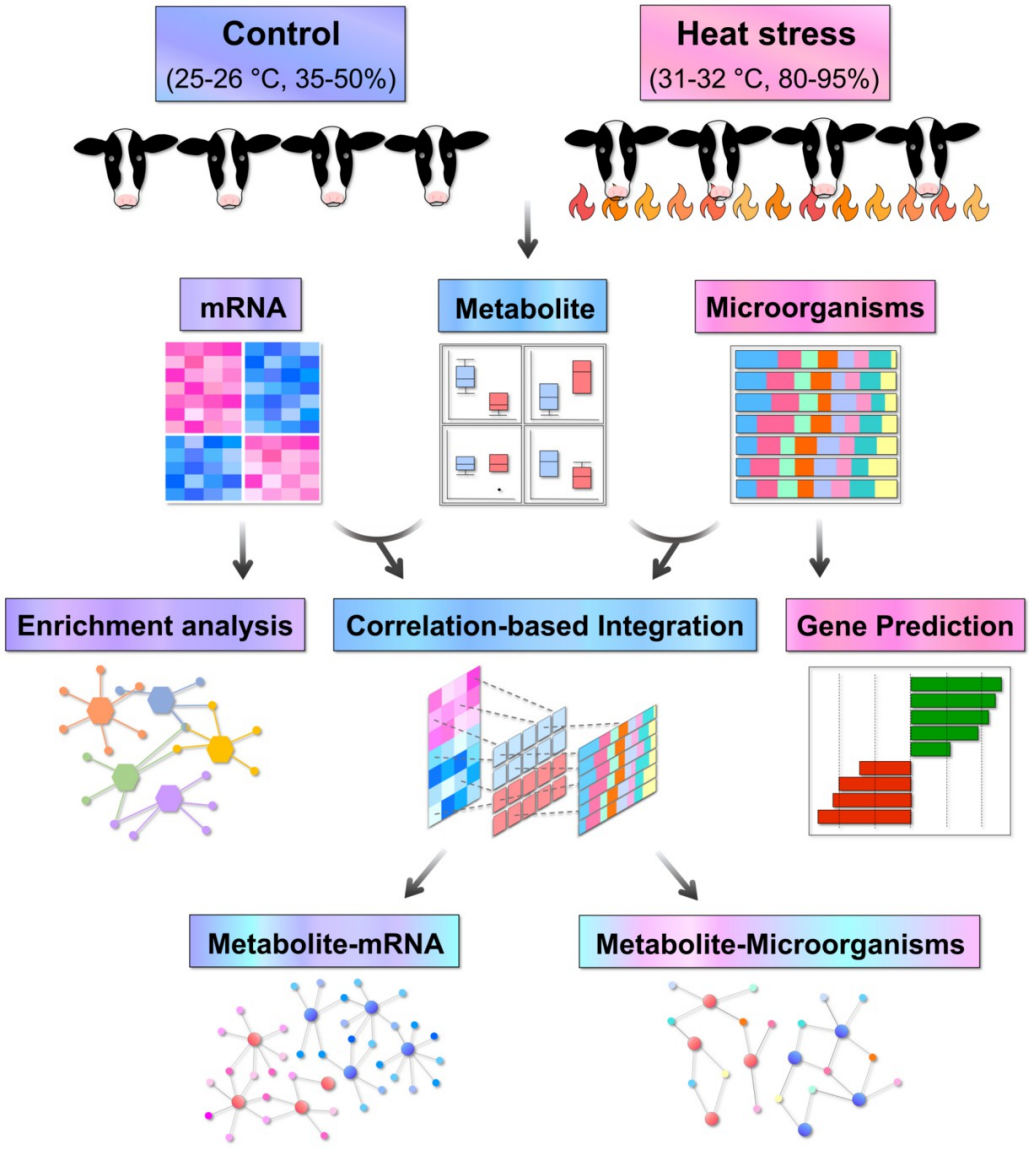
Analytical workflow for this study. Blood and fecal samples from animals were used for metabolite analysis, RNA-seq and 16S rRNA-seq, respectively, and the generated data were analyzed and integrated with their correlation.

The experimental process is illustrated in Fig 1.

### Identification of metabolites in blood

**Fig 2:**
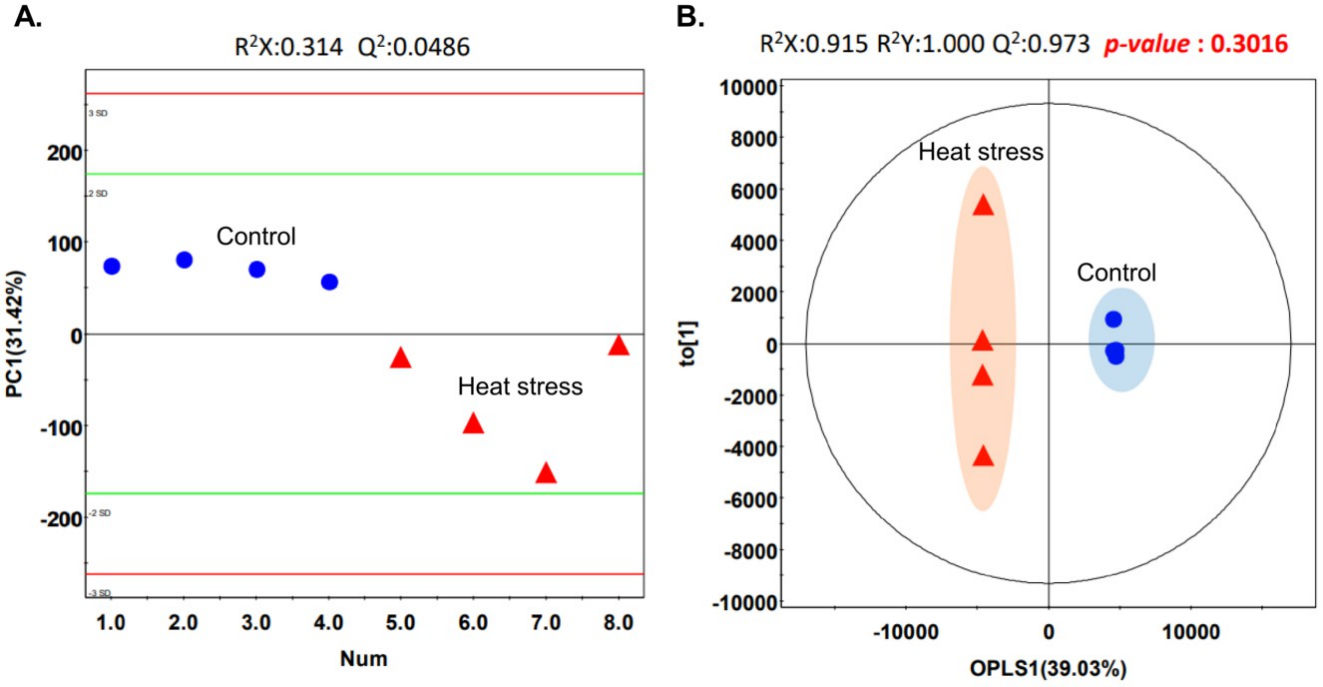
The PCA and OPLS-DA model. (A) PCA plot showing the distance between two groups. (B) Score plot derived from OPLS-DA model of blood samples, tend to be divided between two groups but not significant.

**Fig 3:**
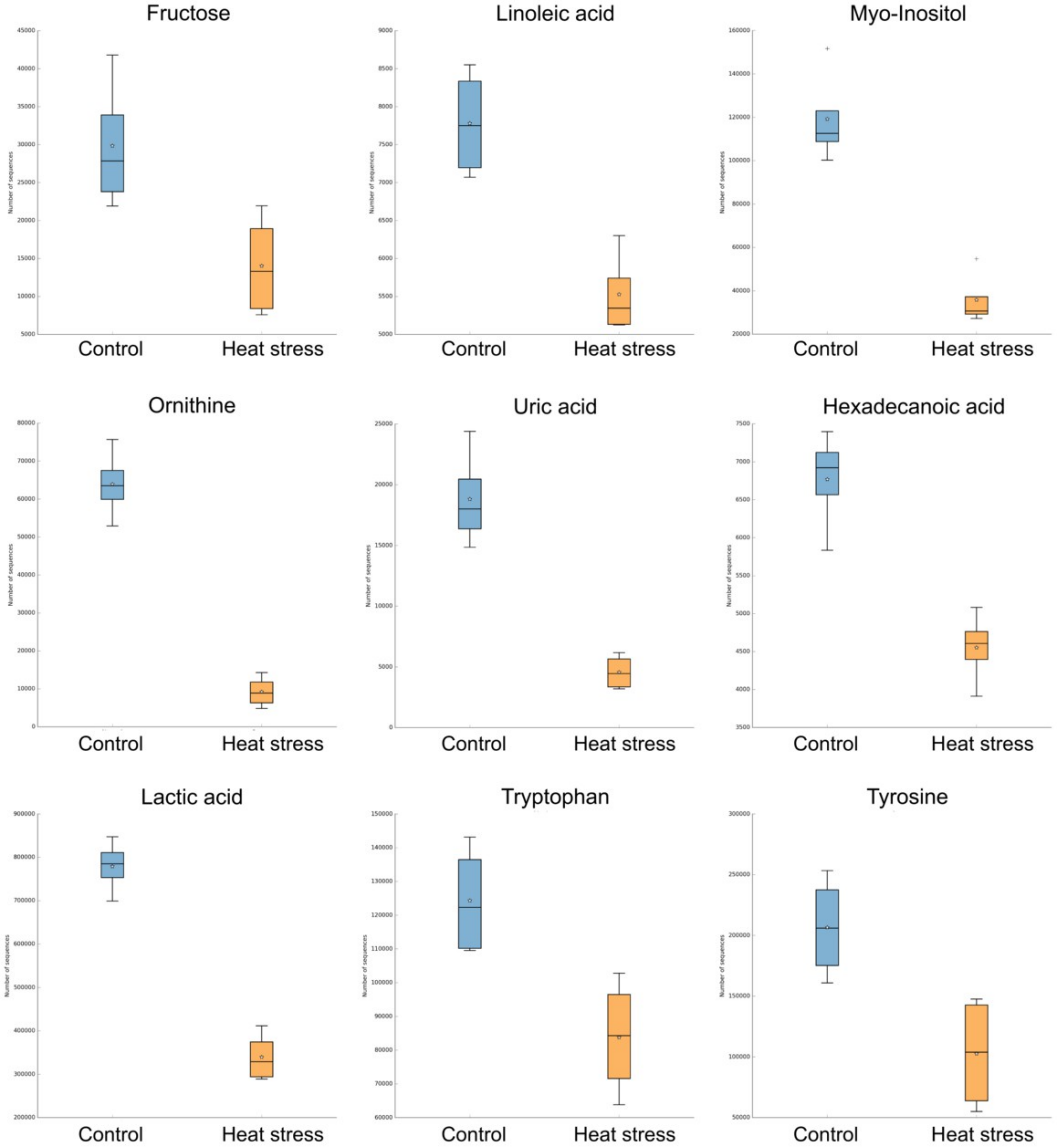
The relative amounts of metabolites. Box plot showing significant metabolites with VIP ≥1 and *p* <0.05, these metabolites reduced in Severe as follows: Ornithine, Tryptophan, and Tyrosine from amino acids, Hexadecanoic acid and Linoleic acid from fatty acids, Fructose and Myo-Inositol from sugar derivatives, and Organic acids from organic acids Lactic acid and uric acid.

To compare the metabolomic analysis results of the two groups, Fig 2 shows the results of principal component analysis (PCA) and orthogonal partial least squares discriminant analysis (OPLS-DA) analyses. No significant difference was found in the OPLS-DA, but there was a tendency to divide the patients into two groups (*p* > 0.05). Of the 29 metabolites, 9 showed significant differences between the two groups. These significant metabolites with variable importance in projection (VIP) ≥ 1 and *p* < 0.05 were ornithine, lactic acid, myoinositol, uric acid, hexadecanoic acid, linoleic acid, tryptophan, tyrosine, and fructose, and the concentrations of metabolites decreased in the high temperature stress group and other metabolites were not significantly different between the two groups. These results are shown in Fig 3, and detailed information, such as sample concentrations is in S1 Table.

### Construction of the raw reads, mapping, and batch correction in RNA seq analysis

**Fig 4:**
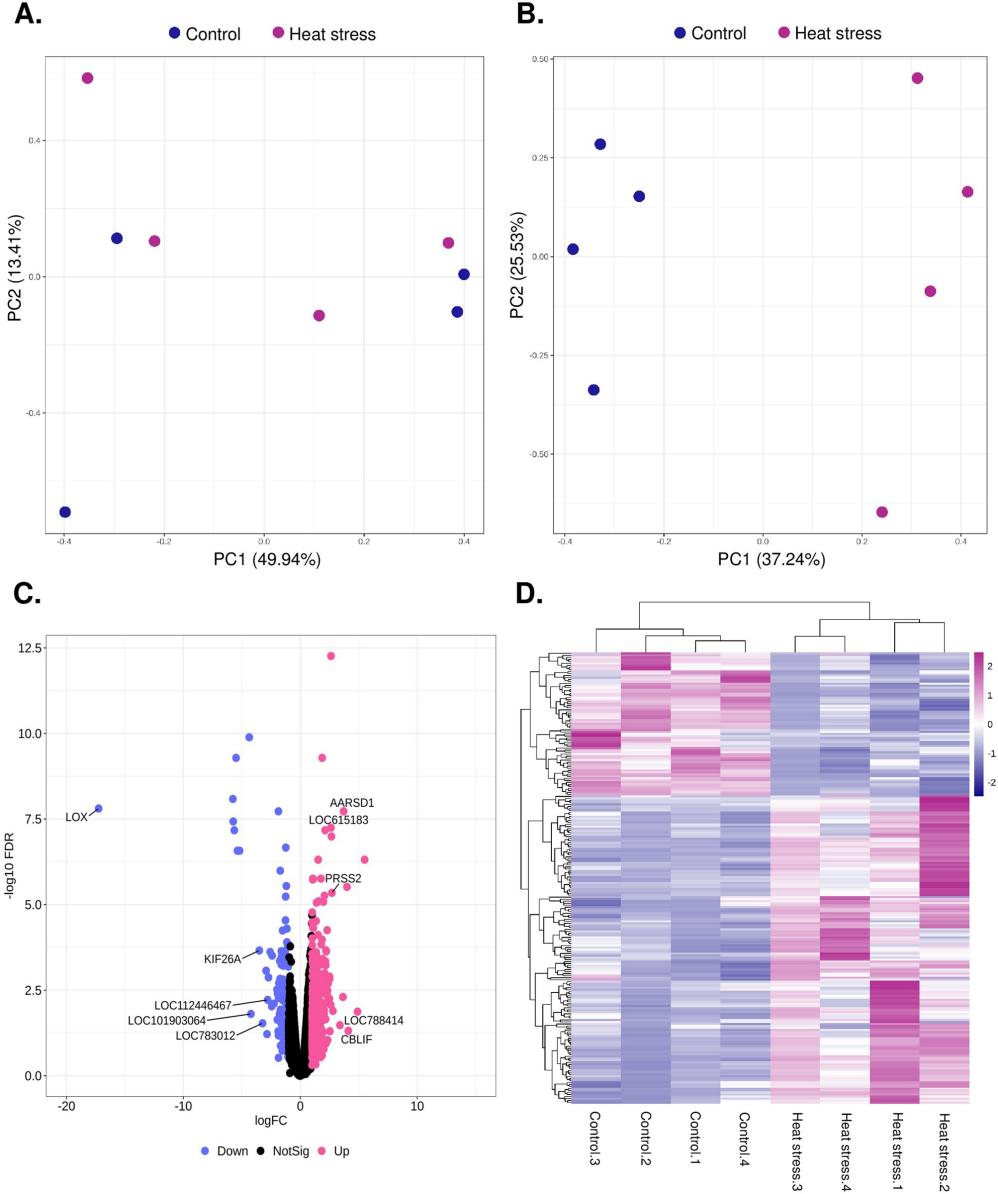
The identification of gene expression patterns and DEGs. (A) and (B) PCA plot showing count data before the batch effect correction and after the removed unwanted factors. (C) Volcano plot showing the up- and down-regulated DEGs removed unwanted factors with FDR < 0.05 and log_2_FC ≥ 1.0 in Severe. (D) Heatmap clustering showing the degrees of DEGs and the clustered samples in two groups, colors indicated pink as up- and blue as down-regulated.

The raw sequence data was generated from 8 blood samples, the data size was 1.8~2.1G, and the GC ratio was 44.76~52.71. Based on the Phred scores, the Q20 and Q30 percentages were 98.03–98.40 and 94.32–95.11. The Hisat2 program was used to map the generated sequences to the reference genome, and 85.75 to 95.38% of the total sequences were mapped for each sample (S2 Table). Between 37.40 and 82.91% of all mapped sequences were assigned to the read-count matrix using the FeatueCounts program. Batch correction was performed using the RUVr function of the RUVSeq R package to remove unwanted elements that interfered with the analysis (Fig 4).

### Identification of DEGs in RNA seq analysis

Based on the read count matrix data, we identified 226 DEGs that met the criteria of FDR < 0.05 and log_2_FC ≥ 1.0 using the edgeR package. Of the 226 DEGs, 154 were upregulated genes and 72 were downregulated genes, we provided detailed information in S3 Table. The top five statistically significant DEGs are shown in Fig 4C, and gene expression patterns are shown by heatmap clustering in Fig 4D. The top five upregulated DEGs were *LOC788414* (epithelial splicing regulatory protein 1), *CBLIF* (cobalamin binding intrinsic factor), *AARSD1* (alanyl-tRNA synthetase domain containing 1), *PRSS2* (serine protease 2) and *LOC615183* (late histone H2B.L4), and the top five down-regulated DEGs are *LOX* (lysyl oxidase), *LOC101903064* (COMM domain-containing protein 6), *KIF26A* (kinesin family member 26A), *LOC783012* (beta-defensin-like) and *LOC112446467* (ubiquitin carboxyl-terminal hydrolase 17-like protein 6) (Table 1). The DEGs in this study included *HSPB8* (heat shock protein family B member 8), *HSPA6* (heat shock protein family A member 6), and *HSPE1* (heat shock protein family E member 1).

**Table 1:** The top 10 DEGs, gene expression level and statistical significance in the heat stress experimental group.

Regulation	Symbol	logFC	FDR
Up	*LOC788414*	4.894	0.013
	*CBLIF*	4.104	0.049
	*AARSD1*	3.702	< 0.001
	*PRSS2*	2.702	< 0.001
	*LOC615183*	2.616	< 0.001
	*COL4A2*	2.529	< 0.05
	*PTGR1*	2.504	0.002
	*ARL4D*	2.483	0.006
	*DEFB4A*	2.333	0.003
	*GATA4*	2.220	0.007
Down	*SRGAP3*	-1.866	< 0.001
	*CYP17A1*	-2.251	0.008
	*LOC112444495*	-2.409	< 0.001
	*ARPIN*	-2.445	0.009
	*LOC112441470*	-2.717	0.001
	*LOC112446467*	-2.792	0.006
	*LOC783012*	-3.227	0.029
	*KIF26A*	-3.503	< 0.001
	*LOC101903064*	-4.223	0.016
	*LOX*	-17.249	< 0.001

*COL4A2*: collagen type IV alpha 2 chain, *PTGR1*: prostaglandin reductase 1, *ARL4D*: ADP ribosylation factor like GTPase 4D, *DEFB4A*: defensin beta 4A, *GATA4*: GATA binding protein 4, *LOC112441470*: steroid 17-alpha-hydroxylase/17,20 lyase, *ARPIN*: actin related protein 2/3 complex inhibitor, *LOC112444495*: steroid 17-alpha-hydroxylase/17,20 lyase, *CYP17A1*: cytochrome P450 family 17 subfamily A member 1, *SRGAP3*: SLIT-ROBO Rho GTPase activating protein 3.

### DEGs enrichment analysis

**Fig 5:**
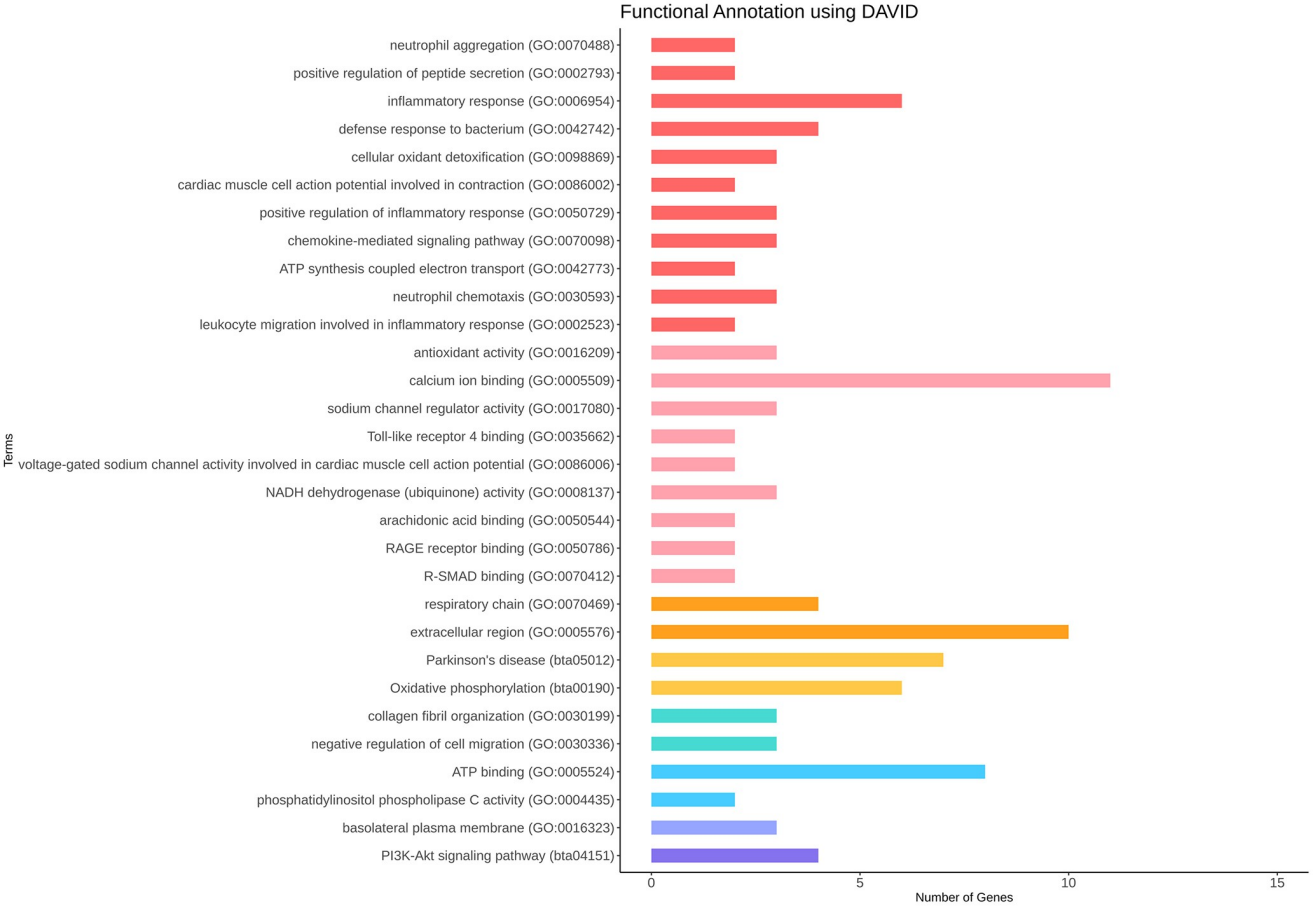
The enriched terms of upregulated DEGs with EASE < 0.1 and *p* < 0.05 in the heat stress experimental group. Colors indicated each category, green (Biological Process), blue (Cellular Component), periwinkle (Molecular Function), and pink (KEGG pathway) in upregulated DEGs. The upregulated DEGs in the heat stress experimental group are considered downregulated DEGs in control group.

**Fig 6:**
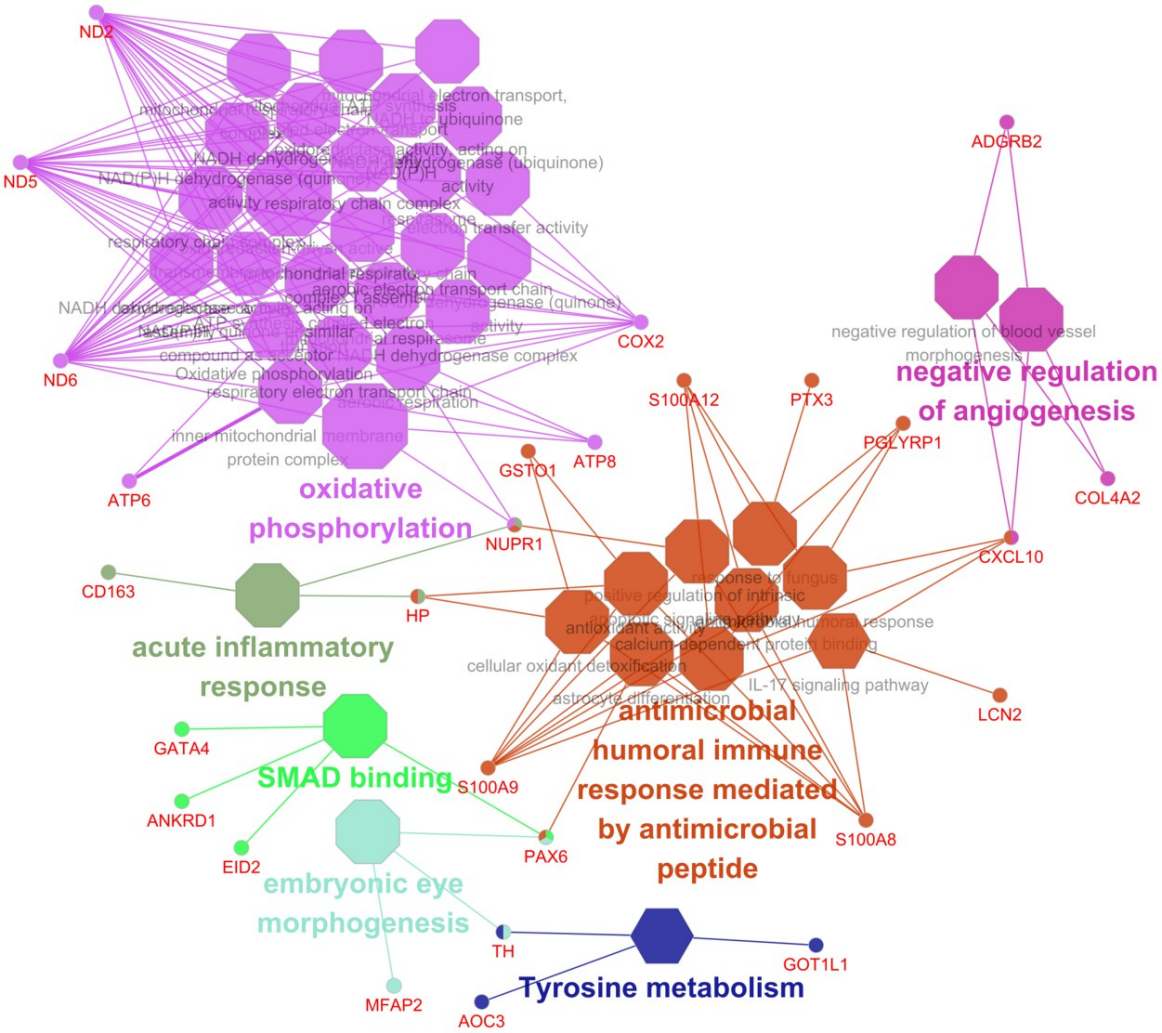
The enriched of upregulated gene network with *p* < 0.05 in the heat stress experimental group. Shapes indicated octagon as GO terms and hexagon as KEGG pathways in upregulated DEGs, and circle as DEGs. The upregulated DEGs in the heat stress experimental group are considered downregulated DEGs in control group.

We performed functional analysis of the upregulated and downregulated DEGs in this study using the gene ontology (GO) and kyoto encyclopedia of genes and genomes (KEGG) pathway databases in ClueGO and DAVID (EASE < 0.1, *p* < 0.05). As shown in the analysis results, up-regulated genes were associated with Oxidative phosphorylation (bta00190), IL-17 signaling pathway (bta04657), antimicrobial humoral immune response mediated by antimicrobial peptide (GO:0061844), antioxidant activity (GO:0016209), calcium ion binding (GO:0005509), and mitochondrion (GO:0005739); The down-regulated genes were associated with collagen fibril organization (GO:0030199), negative regulation of cell migration (GO:0030336) and Cortisol synthesis and secretion (bta04927) (Fig 5). In the ClueGO analysis, the upregulated genes included acute inflammatory response (GO:0002526), IL-17 signaling pathway (KEGG:04657), antimicrobial humoral immune response mediated by antimicrobial peptide (GO:0061844), Oxidative phosphorylation (KEGG:00190), antioxidant activity (GO:0016209) mitochondrial respirasome (GO:0005746), and ATP synthesis coupled electron transport (GO:0042773). The downregulated genes were one-carbon compound transport (GO:0019755) and collagen fibril organization (GO:0030199) (*p* < 0.05). The gene network of these DEGs using ClueGO is shown in Fig 6 and Table 2, and the detailed results can be found in S4 and S5 Tables.

**Table 2:** The enriched of upregulated gene network and the associated with up regulated DEGs in the heat stress experimental group.

ID	Terms	Associated Genes
GO:0002526	acute inflammatory response	*CD163*, *HP*, *NUPR1*
GO:0003954	NADH dehydrogenase activity	*ND2*, *ND5*, *ND6*
GO:0003955	NAD(P)H dehydrogenase (quinone) activity	*ND2*, *ND5*, *ND6*
GO:0005746	mitochondrial respirasome	*COX2*, *ND2*, *ND5*, *ND6*
GO:0005747	mitochondrial respiratory chain complex I	*ND2*, *ND5*, *ND6*
GO:0006119	oxidative phosphorylation	*ATP8*, *COX2*, *ND2*, *ND5*, *ND6*, *NUPR1*
GO:0006120	mitochondrial electron transport, NADH to ubiquinone	*ND2*, *ND5*, *ND6*
GO:0008137	NADH dehydrogenase (ubiquinone) activity	*ND2*, *ND5*, *ND6*
GO:0009055	electron transfer activity	*COX2*, *ND2*, *ND5*, *ND6*
GO:0009060	aerobic respiration	*ATP8*, *COX2*, *ND2*, *ND5*, *ND6*, *NUPR1*
GO:0009620	response to fungus	*PGLYRP1*, *PTX3*, *S100A12*
GO:0010257	NADH dehydrogenase complex assembly	*ND2*, *ND5*, *ND6*
GO:0015453	oxidoreduction-driven active transmembrane transporter activity	*COX2*, *ND2*, *ND5*, *ND6*
GO:0016209	antioxidant activity	*GSTO1*, *HP*, *S100A8*, *S100A9*
GO:0016525	negative regulation of angiogenesis	*ADGRB2*, *COL4A2*, *CXCL10*
GO:0016651	oxidoreductase activity, acting on NAD(P)H	*ND2*, *ND5*, *ND6*
GO:0016655	oxidoreductase activity, acting on NAD(P)H, quinone or similar compound as acceptor	*ND2*, *ND5*, *ND6*
GO:0019646	aerobic electron transport chain	*ND2*, *ND5*, *ND6*
GO:0019730	antimicrobial humoral response	*CXCL10*, *PGLYRP1*, *S100A12*, *S100A9*
GO:0022904	respiratory electron transport chain	*COX2*, *ND2*, *ND5*, *ND6*
GO:0030964	NADH dehydrogenase complex	*ND2*, *ND5*, *ND6*
GO:0032981	mitochondrial respiratory chain complex I assembly	*ND2*, *ND5*, *ND6*
GO:0042773	ATP synthesis coupled electron transport	*COX2*, *ND2*, *ND5*, *ND6*
GO:0042775	mitochondrial ATP synthesis coupled electron transport	*ND2*, *ND5*, *ND6*
GO:0045271	respiratory chain complex I	*ND2*, *ND5*, *ND6*
GO:0046332	SMAD binding	*ANKRD1*, *EID2*, *GATA4*, *PAX6*
GO:0048048	embryonic eye morphogenesis	*MFAP2*, *PAX6*, *TH*
GO:0048306	calcium-dependent protein binding	*S100A12*, *S100A8*, *S100A9*
GO:0048708	astrocyte differentiation	*PAX6*, *S100A8*, *S100A9*
GO:0050136	NADH dehydrogenase (quinone) activity	*ND2*, *ND5*, *ND6*
GO:0061844	antimicrobial humoral immune response mediated by antimicrobial peptide	*CXCL10*, *PGLYRP1*, *S100A12*, *S100A9*
GO:0070469	respirasome	*COX2*, *ND2*, *ND5*, *ND6*
GO:0098800	inner mitochondrial membrane protein complex	*ATP6*, *ATP8*, *COX2*, *ND2*, *ND5*, *ND6*
GO:0098803	respiratory chain complex	*COX2*, *ND2*, *ND5*, *ND6*
GO:0098869	cellular oxidant detoxification	*GSTO1*, *HP*, *S100A8*, *S100A9*
GO:2000181	negative regulation of blood vessel morphogenesis	*ADGRB2*, *COL4A2*, *CXCL10*
GO:2001244	positive regulation of intrinsic apoptotic signaling pathway	*NUPR1*, *S100A8*, *S100A9*
KEGG:00190	Oxidative phosphorylation	*ATP6*, *ATP8*, *COX2*, *ND2*, *ND5*, *ND6*
KEGG:00350	Tyrosine metabolism	*AOC3*, *GOT1L1*, *TH*
KEGG:04657	IL-17 signaling pathway	*CXCL10*, *LCN2*, *S100A8*, *S100A9*

### Construction of the raw reads and result of assembly

The raw sequence data consisted of 192,646–230,178 reads from eight fecal samples, with GC percentages ranging from 52.36 53.39. Based on the Phred score, the Q20 and Q30 percentages ranged from 93.72 to 94.45 and 86.26 to 87.33. In the assembly of read sequences for operational taxonomic units (OTUs) identification, 85,140 to 112,388 reads were used, with their GC percentages ranging from 51.97 to 53.11 and the proportions of leads above Q20 and Q30 ranged from 98.66 to 98.86 and 95.63 to 96.06, respectively. Through this, 18,879 25,795 OUTs were discovered, and detailed information can be found in S6 Table.

### Gut microbiome taxonomy analysis

**Fig 7:**
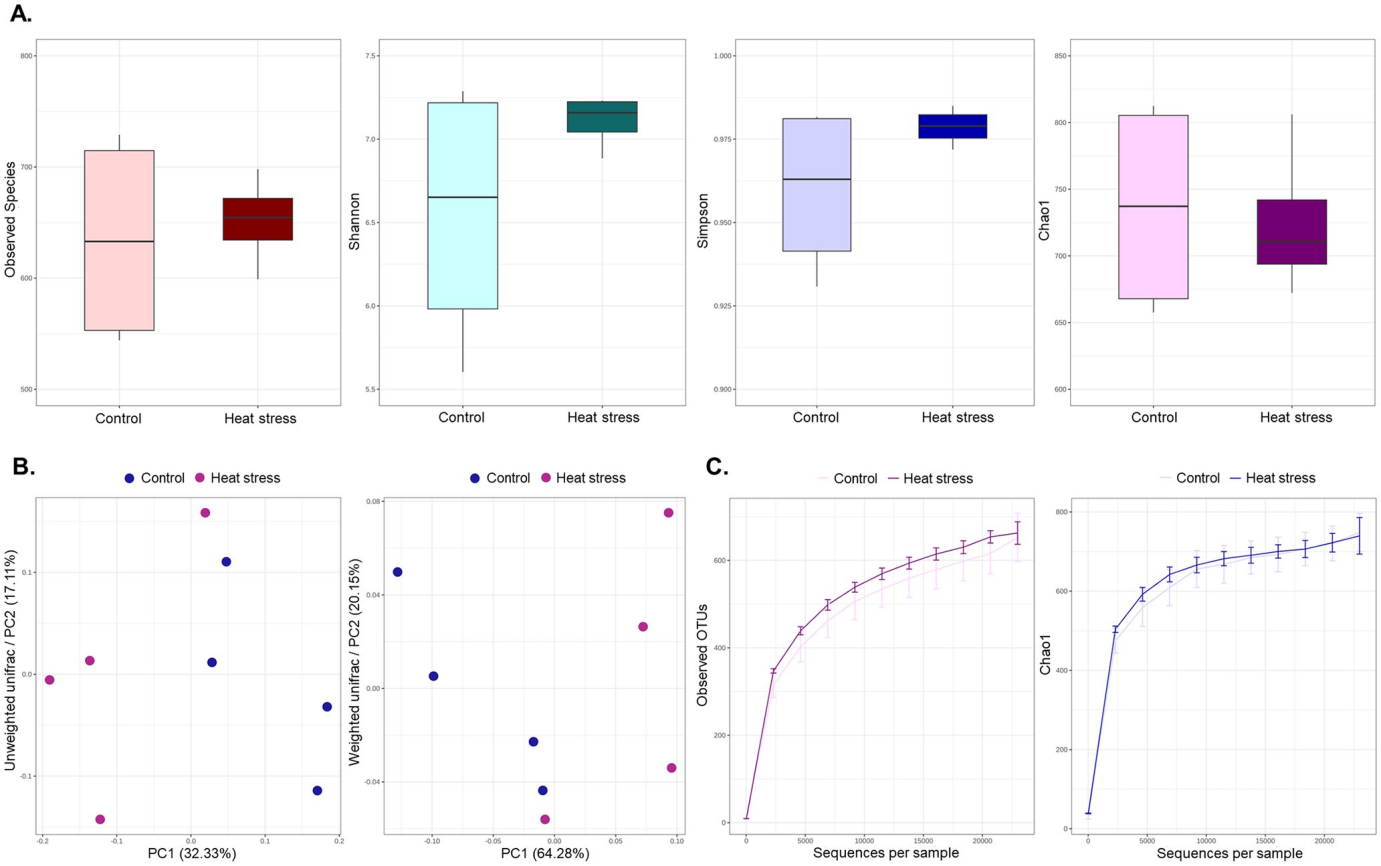
Alpha, beta diversity metrics and alpha rarefaction of microbial communities. (A) Alpha diversity showing Observed species, Shannon, Simpson, and Chao1 index. (B) Beta diversity showing distance of samples through unweighted and weighted UniFrac. (C) The generated alpha rarefaction curves showing sampling depth.

**Fig 8:**
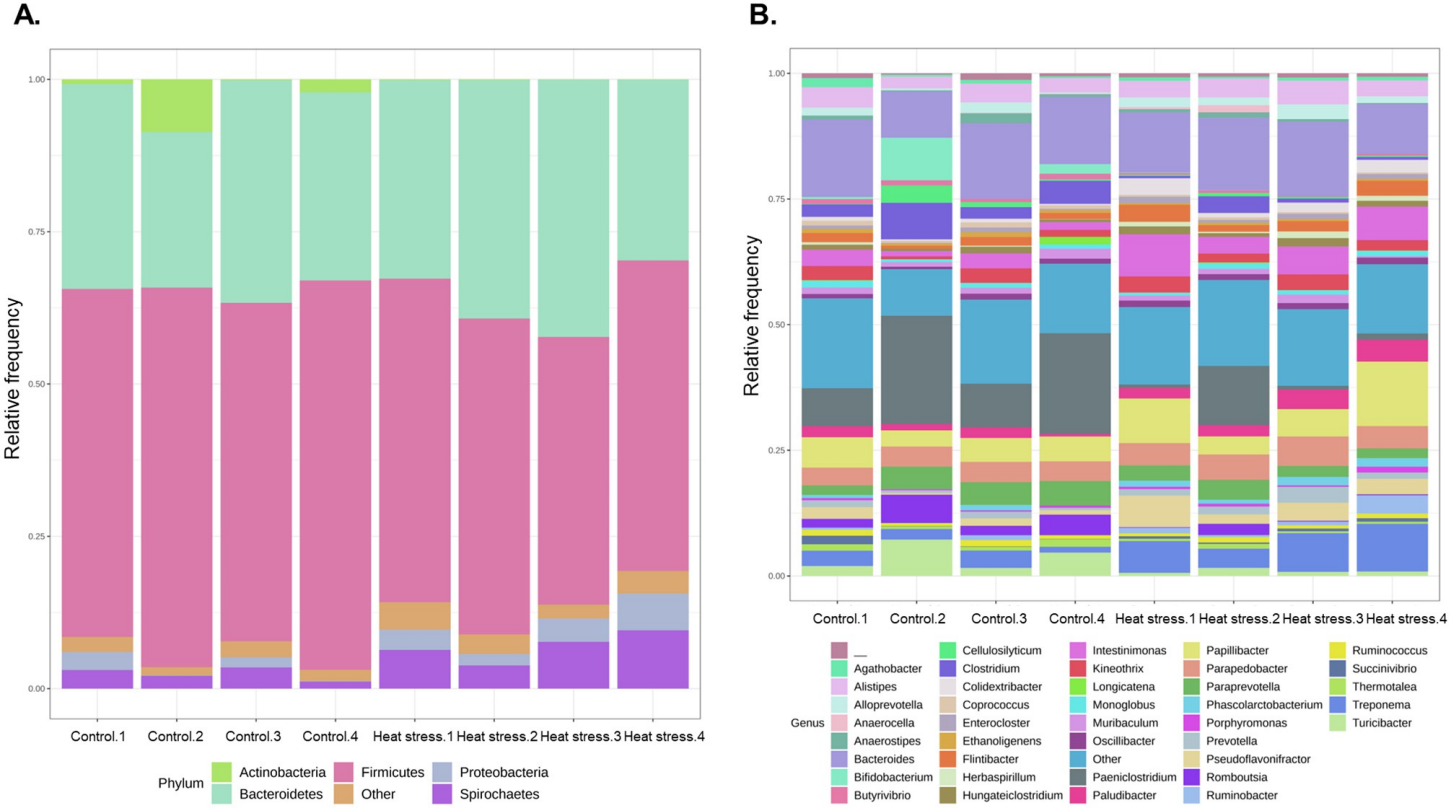
Relative abundance. (A) phylum level. (B) genus level. Each color indicated taxa, respectively, taxon sequences were assigned to OTUs in each sample, and abundant taxa more than 1% indicated their name whereas <1% was included in the other on the plots.

**Fig 9:**
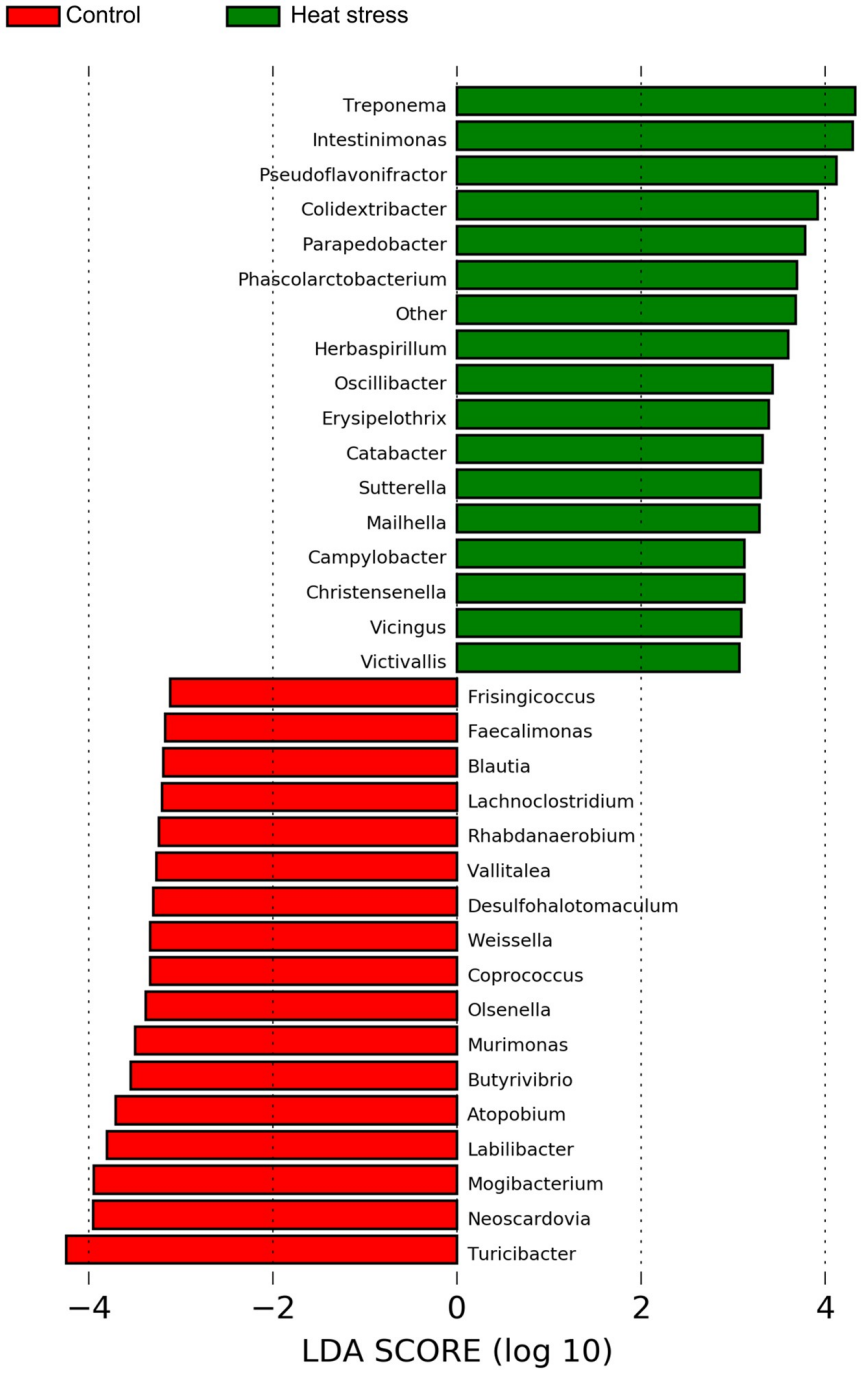
Microbial abundance showing significant difference at genus level. The characterized genus were compared significantly with at linear discriminant analysis (LDA) ≥ 2.

We estimated alpha diversity metrics to compare the abundances of the two groups of species and could not confirm any significant difference between the two groups in Chao1, observed OTUs, and the PD whole tree (Fig 7). We also checked the beta diversity metrics and sampling depth using weighted (quantitative), unweighted, and alpha rarefaction (qualitative) methods in Fig 7. Taxonomy assignment was performed based on the NCBI 16S rRNA database. In the taxonomic analysis, Actinobacteria, Bacteroidetes, Firmicutes, Proteobacteria, and Spirochaetes at the phylum level and *Bacteroides*, *Paeniclostridium*, *Papillibacter*, *Treponema* and *Parapedobacter* at the genus level were identified as the main microorganisms. (Fig 8). At the genus level, the differentially abundant microorganisms between the two groups were identified as *Turicibacter*, *Neoscardovia*, and *Mogibacterium* in the control group and *Treponema*, *Intestinimonas*, and *Pseudoflavonifractor* in the high-temperature stray group, as shown in Fig 9 and Table 3. Additionally, the total microbial abundance information is provided in S7 and S8 Tables.

**Table 3:** Top 10 genus of microbial differential abundance.

Groups	Genus	LDA
Control	*Turicibacter*	4.244
	*Neoscardovia*	3.955
	*Mogibacterium*	3.947
	*Labilibacter*	3.805
	*Atopobium*	3.709
	*Butyrivibrio*	3.542
	*Murimonas*	3.496
	*Olsenella*	3.380
	*Coprococcus*	3.336
	*Weissella*	3.331
Heat stress	*Treponema*	4.324
	*Intestinimonas*	4.301
	*Pseudoflavonifractor*	4.123
	*Colidextribacter*	3.919
	*Parapedobacter*	3.780
	*Phascolarctobacterium*	3.693
	Other	3.679
	*Herbaspirillum*	3.596
	*Oscillibacter*	3.432
	*Erysipelothrix*	3.387

### Predicted functional profiles of metagenome

**Fig 10:**
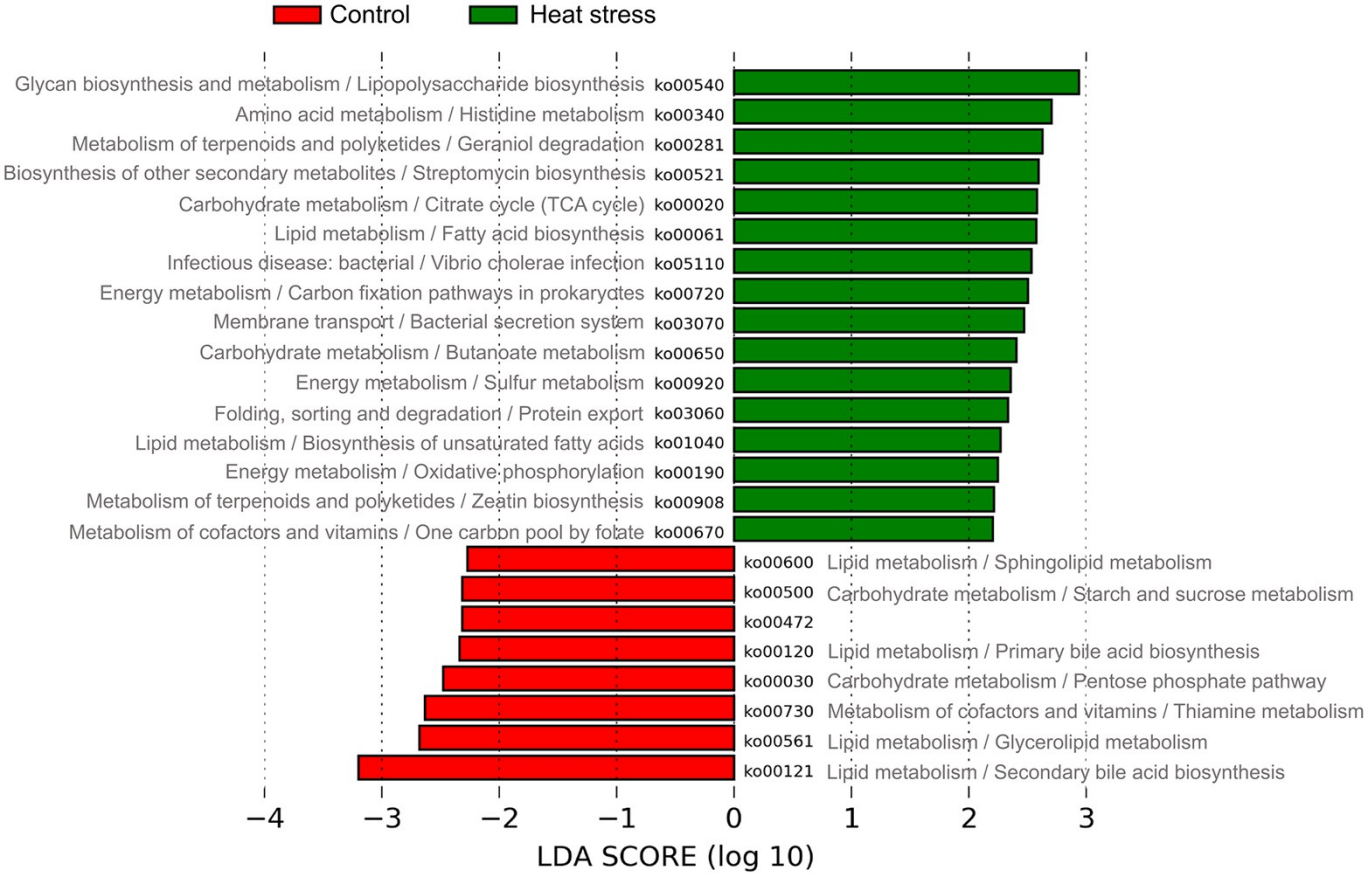
The predicted functions of microbial genome. The functional pathways of microbial communities were compared significantly with LDA ≥ 2.

We extracted the KEGG KO numbers and unstratified abundance tables from the PICRUSt2 analysis and confirmed their statistical significance using the LEfSe program. The functional genomes of microorganisms with higher expression levels in the control group were associated with secondary bile acid biosynthesis (ko00121), glycerolipid metabolism (ko00561), thiamine metabolism (ko00730), the pentose phosphate pathway (ko00030), and primary bile acid biosynthesis (ko00120) and were associated with high-temperature stress. Such genomes that were highly expressed in the experimental group were involved in lipopolysaccharide biosynthesis (ko00540), histidine metabolism (ko00340), geraniol degradation (ko00281), streptomycin biosynthesis (ko00521), and citrate cycle (TCA cycle) (ko00020). This information is shown in Fig 10 and Table 4; detailed information is provided in S9 Table.

**Table 4:** Microbial genome predictions and statistical analysis.

Groups	ID	Pathway	LDA
Control	ko00121	Secondary bile acid biosynthesis	3.198
	ko00561	Glycerolipid metabolism	2.683
	ko00730	Thiamine metabolism	2.632
	ko00030	Pentose phosphate pathway	2.478
	ko00120	Primary bile acid biosynthesis	2.339
	ko00472	(Unclear)	2.315
	ko00500	Starch and sucrose metabolism	2.315
	ko00600	Sphingolipid metabolism	2.273
Heat stress	ko00540	Lipopolysaccharide biosynthesis	2.938
	ko00340	Histidine metabolism	2.705
	ko00281	Geraniol degradation	2.630
	ko00521	Streptomycin biosynthesis	2.596
	ko00020	Citrate cycle (TCA cycle)	2.580
	ko00061	Fatty acid biosynthesis	2.576
	ko05110	Vibrio cholerae infection	2.535
	ko00720	Carbon fixation pathways in prokaryotes	2.503
	ko03070	Bacterial secretion system	2.470
	ko00650	Butanoate metabolism	2.402
	ko00920	Sulfur metabolism	2.359
	ko03060	Protein export	2.331
	ko01040	Biosynthesis of unsaturated fatty acids	2.270
	ko00190	Oxidative phosphorylation	2.245
	ko00908	Zeatin biosynthesis	2.213
	ko00670	One carbon pool by folate	2.204

### Correlation network containing multi-omics data

**Fig 11:**
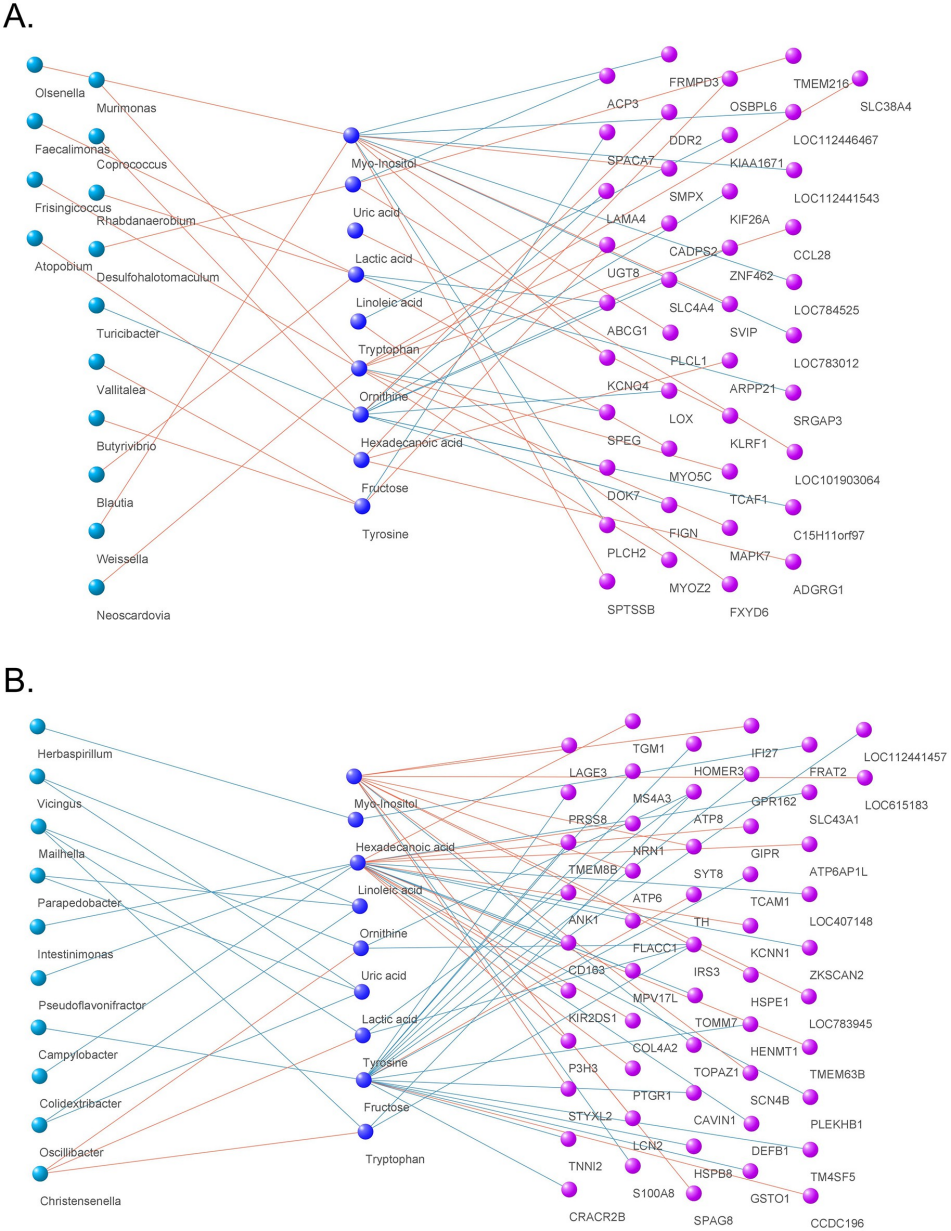
Significant correlation network between metabolome, transcriptome, and microbiome. The correlation network is considered *p* <0.05 and *r*^2^ ± ≥ 0.95 with Spearman’s method. (A) control group. (B) heat stress experimental group.

We identified significant correlations between metabolite-microorganisms and metabolite-upregulated DEGs in two groups. These correlations consisted of 14 microbes and 41 genes in the control group, and 17 microbes and 56 genes in the heat stress group. Additionally, more negative correlations were observed in the high-temperature stress group than in the control group. (Heat Stress Group: 47, Control: 21). Among the metabolites, Myo-Inositol, Ornithine, and Hexadecanoic acid in the control group, and Myo-Inositol, Linoleic acid, and fructose in the high-temperature stress group were closely related to the DEG. The network using the integrated correlation information is shown in Fig 11, and detailed information can be found in S10 Table.

## Discussion

In this study, metabolite changes, gene expression patterns, microbial community changes, and their correlations with heat stress in dairy cows were investigated. One experimental group placed under high-temperature stress for seven days in a high humidity environment compared to the control group. Next, metabolomic and transcriptome analyses of blood and intestinal microbiomes were performed using cattle feces. All data were integrated through correlation analysis, and the potential changes caused by high-temperature stress were analyzed.

### Altered concentration of blood metabolites under heat stress condition

In this study, the comparative analysis of metabolites between the control group and the high-temperature stress experimental group, among a total of 29 metabolites, nine metabolites (Ornithine, Tryptophan, and Tyrosine from amino acids, Hexadecanoic acid and Linoleic acid from fatty acids, Fructose and Myo-Inositol from sugar derivatives, and Lactic acid and uric acid from organic acid) showed statistical differences (VIP ≥1, *p* <0.05). As shown in previous studies, the metabolome can also be changed by heat, and the fact that this can occur in various tissues was an important basis for our study [50]. Previous studies have reported that heat stress can affect some blood metabolites (ornithine, tryptophan, and tyrosine) that decreased in the heat stress experimental group [51, 52]. The thermal environment can inhibit protein synthesis, thereby reducing protein production and affecting protein metabolism [53]. We confirmed that our findings overlapped with those of previous studies on amino acid metabolites. Amino acids are an important source for protein synthesis and can be adversely affected by environmental factors such as temperature and humidity [30, 54]. Controlled amino acid synthesis is expected to reduce the production of low-quality livestock products by decreasing protein synthesis [55]. The decrease in protein synthesis caused by heat stress prevents the generation of mutated proteins, and relative oxygen species (ROS) derived from oxidative stress induced by heat stress maintain cell homeostasis by altering the metabolic system [56]. In addition, previous studies have reported that heat stress can downregulate blood metabolites, and the present study showed that blood metabolites, including fructose, were reduced in the heat stress experimental group [57, 58]. In general, stressful conditions induce fructose synthesis in the body, which increases oxidative stress due to excessive ROS production whereas this result indicated to be reduced in the heat-stressed experimental group of dairy cows [28]. Fructose can be utilized as energy source and depleted ATP due to the rapid phosphorylation, and reduced fructose amount is thought to lead activation of pathways related to energy metabolism [59–62]. However, these findings are different from recent studies and not supported to be modulated by heat stress condition with lacking evidence. Therefore, this is only explained to be modulated in amounts of blood metabolites and associated with upregulated gene expression and is ambiguous that response to heat stress with only reduced fructose. On the other hand, linoleic acid, an essential fatty acid in animals, is known to promote inflammation and protect against oxidative stress, and a decrease in linoleic acid in individuals exposed to high-temperature stress is thought to help maintain homeostasis [25, 26]. In addition, fatty acids, sugar derivatives, and organic acids were significantly reduced in the high-temperature stress experimental group. Uric acid is a useful marker that acts as an antioxidant against oxidative damage, and its concentration is generally expected to increase under heat stress conditions. A previous study reported that the modulation of blood metabolites is considered an important process to acclimatize, especially, is accelerated cellular energy depletion under heat stress condition [62]. However, previous studies have shown that amino acid concentrations decrease under chronic heat stress [20, 27, 57]. However, in this study, the mechanism of the change in metabolite concentrations due to heat stress could not be completely explained because there were no detailed physiological data for each individual. Furthermore, sample size is not large enough, four samples in each group are not representative of the entire population, thus, it should be considered limited in this experiment. In future studies, it is necessary to measure various physiological and biochemical data such as heart rate, body temperature, feed intake, daily weight gain, milk production, and hormones, and previous studies documented that heart rate, body temperature and cortisol are shown increased, and also, feed intake, daily weight gain, milk production, and triiodothyronine are shown reduced, which can contribute to reduction of heat production, leading to adaptation under heat stress conditions [21, 62, 63]. However, considering the homeostasis mechanism of the individual, heat stress had a clear effect on the concentrations of amino acids, fatty acids, sugar derivatives, and organic acids in the blood. We believe that the reduction of fructose and linoleic acid are related to cellular energy metabolism and immune system by heat stress condition, which are highly likely to be modified metabolites for cellular homeostasis.

### Changed gene expression pattern and their function by heat stress

We performed a transcriptomic analysis of eight samples (four control groups and four experimental groups) and detected 226 DEGs, including protein-coding genes such as *HSPB8*, *HSPA6* and *HSPE1*. Of the 226 DEGs, 154 and 72 genes were upregulated and downregulated, respectively, in the heat stress experimental group. We then performed functional analysis of the DEGs using the GO and KEGG databases. Oxidative phosphorylation (bta00190), IL-17 signaling pathway (bta04657), antimicrobial humoral immune response mediated by antimicrobial peptide (GO:0061844), and NADH dehydrogenase (ubiquinone) activity (GO:0008137) were found in the upregulated gene group, and collagen fibril organization was found in the downregulated gene group. (GO:0030199) were confirmed to be statistically significant in both databases. The IL-17 signaling pathway (KEGG:04657) and the antimicrobial humoral immune response mediated by antimicrobial peptides (GO:0061844) shared the genes including *CXCL10* (C-X-C motif chemokine ligand 10) and *S100A9* (S100 calcium binding protein A9). In addition, mitochondrion (GO:0005739) and oxidative phosphorylation (bta00190) also shared genes including *ATP6* (ATP synthase F0 subunit 6), *COX2* (cytochrome c oxidase subunit II), *ND5* (NADH dehydrogenase subunit 5), and *ND6* (NADH dehydrogenase subunit 6), which is expressed in mitochondria.

Among the significant pathways identified in the functional analysis, the IL-17 (Interleukin-17) signaling pathway also consists of the IL-17A to IL-17F family genes. These genes may be involved in tissue homeostasis, inflammatory responses, and immune defense and protect the host against microbial invasion [64, 65]. In addition, IL-17, a cytokine involved in the production of antimicrobial peptides to defend the host against inflammatory diseases, increases under oxidative and heat stress [66]. In addition, *CXCL10*, *CCL16* (C-C motif chemokine ligand 16), and *S100A9* are associated with antimicrobial humoral immune responses, and previous studies have shown that overexpression of IL-17 generates CXCL, which regulates inflammation as a chemokine [67, 68]. Chemokines secrete chemotactic signals that directly activate immune cells in specific tissues and play an important role in immune responses [69]. *S100A9* also contributes to antimicrobial peptide production for host defense and is associated with increased activation of the IL-17 signaling pathway [70]. Previous studies have reported that heat stress environments can induce host defenses by altering gene expression patterns related to immune function, triggering highly activated IL-17 signaling pathways via related genes, and inducing chemokines in T cells. The upregulated DEGs, *LCN2* (lipocalin 2), *PGLYRP1* (peptidoglycan recognition protein 1), *CD163* (CD163 molecule), *CXCL10*, and *CCRL2* (C-C motif chemokine receptor like 2), are involved in innate immune, which is affected by heat stress, and, thus, leading to activation of immune response with cellular oxidation and inflammation for maintaining homeostasis [14, 63, 71]. Therefore, heat stress condition may contribute to modulated genes related to immune response. We believe that this immune response is useful for protecting against inflammation caused by heat stress [72–74]. However, various factors in the immune response can change with age, genetic characteristics, and species, and the mechanism of IL-17 activation in dairy cows has not yet been fully elucidated [73, 75]. In addition, *HSPA6*, *HSPB8*, and *HSPE1* regulate protein quality in response to heat stress, maintain the immune system, and maintain mitochondrial function to protect cells from stress. In this study, it was also observed that the expression level of these genes increased in the high-temperature stress experimental group [13, 76, 77]. In particular, the HSP70 gene family, to which the upregulated *HSPA6* in the experimental group belongs, can interact with other molecular chaperones, prevent extracellular infection, repair, and degrade proteins that are misfolded, aggregated, and denatured by heat stress. They play important roles in protein quality control [93, 94]. *HSPB8*, which belongs to the HSP22 gene family and is a member of the small heat shock protein (sHSP) family, is upregulated when exposed to stressful conditions, accelerating the efficient repair and degradation of damaged proteins [78]. In addition, it was confirmed that oxidative phosphorylation associated with *ATP6*, *ATP8*, *COX2*, *ND2*, *ND5*, and *ND6* was an important pathway with mitochondrion term in the heat-stress experimental group. Oxidative phosphorylation in the mitochondria is a key process in ATP synthesis and plays a role in free radical generation and cell death [79]. Previous studies have shown that upregulated HSPs are mitochondrial chaperones that may be related to mitochondrial increases and related gene expression in response to reduced oxidative phosphorylation under various stress conditions, thereby maintaining cellular homeostasis [80–82]. In addition, it is known that maintaining oxidative phosphorylation in a stressful environment does not depend only on HSPs and can be protected by the mitochondrial antioxidant system via *ND2*, *ND5*, and *ND6* [83]. Moreover, recent evidence showed that HSPs indicated to interact with immune system, modulate inflammation, and also, these tend to increase with cytokines under various stress conditions, which leads to maintenance of immune homeostasis [84, 85]. We identified upregulated HSPs and cytokine activation pathways, and such findings suggest that heat stress condition may induce immune response. NADH is essential for redox reactions in organisms, provides electrons for oxidative phosphorylation, contributes to reductive biosynthetic processes of fatty acids and nucleic acids, and is normally maintained at moderate levels with pro-oxidants [86]. However, overproduction of NADH greatly induces reduction stress and oxidative phosphorylation for the NADH/NAD^+^ imbalance, resulting in oxidative stress due to excessive ROS production in the mitochondria [87, 88]. Therefore, additional experiments are required to confirm the role of NADH-related genes in the heat-stress group. Based on previous studies, it is possible to infer that the oxidative phosphorylation of HSP- and NADP-related genes contributes to the maintenance of homeostasis under heat stress conditions [89–92]. Through transcriptome analysis, we found that heat stress altered the gene expression patterns related to inflammatory responses and cellular energy production in cow blood cells. However, these findings were performed using four samples in each group, which are limited discussion in more general results with lacking sample size, therefore, interpretation of such results should be carefully considered. These gene expression changes are thought to occur during reactions related to protein protection, immune system establishment, cellular homeostasis, and oxidative stress caused by mitochondrial NADH. We believe that these results will be helpful for the theoretical understanding of the biological functions altered by cow heat stress.

### Composition of microbial abundance and their function under heat stress condition

We produced 16s rRNA data and performed analyses using fecal samples to study the intestinal microbiota of dairy cows participating in high-temperature stress experiments. We used 16s rRNA data to estimate microbial diversity statistics, create an OTU table, identify microorganisms that showed significant differences between the control and experimental groups, and perform functional predictive analysis for important microorganisms based on the KEGG pathway database. As a result, at the genus level, significantly more microorganisms were identified in 17 in both the control and the heat stress experimental group, and 8 in the control group and 16 in the case of heat stress showed significant biological pathways with LDA ≥ 2.0.

In the analysis of alpha diversity metrics in microorganisms, no statistical difference was observed between the two groups; however, in the analysis of beta diversity metrics, the two groups tended to be clearly divided. In the high-temperature stress experimental group, *Intestinimonas* and *Pseudoflavonifractor* were differentially more abundant than in the control group. It is known to produce butyrate, one of the major microbial metabolites, by using amino acids and amino acids [93, 94]. Butyrate plays an important role in controlling inflammatory responses and inhibits oxidative stress and proteolysis [95]. Various studies have shown that butyrate indirectly contributes to energy metabolism in the host by regulating NADH/NAD^+^ balance and ATP levels [96]. Butyrate can also help maintain intestinal mucosal integrity and tissue homeostasis in a thermal environment [97, 98]. Therefore, we believe that increasing butyrate-producing *Intestinimonas* and *Pseudoflavonifractor* could protect the intestinal tissue and function in response to heat stress. In addition, functional analysis identified important pathways of major microorganisms in the gut microbiota of subjects in the high-temperature stress test group at the KEGG level 3. Lipopolysaccharide biosynthesis (ko00540), an important pathway in the high-temperature stress experimental group, is a major component of the outer membrane of gram-negative bacteria and is known to induce and interact with host immune responses [99]. In particular, low concentrations of lipopolysaccharide regulate immune activity against pathogen infections, but high concentrations of lipopolysaccharide cause bacterial and viral infections, leading to septic shock and death. This is considered in [100]. In addition, it was confirmed that butanoate metabolism (ko00650) was closely related to the intestinal microflora of subjects in the high-temperature stress test group, and this pathway is known to be involved in butyrate production [101]. As noted above, butyrate produced by the gut microbiota has been reported to repair intestinal damage and prevent pathogen invasion and is known to play an important role in intestinal homeostasis, especially in a heat stress environment [97, 102]. In addition, microbial butyrate can directly contribute to intestinal immunity for host intestinal homeostasis and has been suggested that influence to host immune response associated with inflammation process [103]. Our analysis indicated that microbe productive butyrate and activation of butanoate metabolism pathway can be induced by heat stress condition, which may alter microbial composition associated with response to heat stress, and it is involved in intestinal immune homeostasis. Interestingly, oxidative phosphorylation (ko00190) was also observed as an important pathway in the functional analysis of intestinal microbial flora, as was the result of the transcriptome analysis of the host. Oxidative phosphorylation by the gut microbiome is related to energy production, utilizing bacterial butyrate as an energy source and accelerating the NADH/NAD^+^ ratio and ATP synthesis in the mitochondria to maintain cellular energy [96, 104]. Similar to transcriptome analysis, excessive oxidative phosphorylation inevitably increases the accumulation of ROS, accelerates the oxidative chain reaction, and decreases the efficiency of oxidative phosphorylation with increasing temperature, resulting in oxidative stress [103, 105]. Among the identified pathways, the citrate cycle (TCA cycle) (ko00020), which is part of the ATP synthesis process, is thought to help maintain homeostasis by reducing oxidative stress through increased activity [99, 105]. In addition, the TCA cycle has been reported to regulate the redox state of cells and limit the production of NADH used in oxidative phosphorylation processes, which may help maintain mitochondrial composition and function during oxidative stress [106, 107]. However, these findings can only be observed in the gut microbiome that can be observed in this study. Although previous studies have reported that the gut microbiome interacts with the host intestine and that heat stress can alter the gut microbiota and metabolic processes, the detailed mechanisms remain unknown [71, 95, 102, 108]. The sample size should be also considered four samples in each group and our results are limited description for statistical significance. In conclusion, we showed that a heat stress environment could alter the gut microbiome composition and differentially enrich pathways related to the immune response, butyrate production, gut homeostasis, and mitochondrial activity (oxidative phosphorylation), thus, heat stress condition induced alternation of microbial composition associated with butyrate-producing bacteria, pathway enrichment related to cellular energy and butyrate metabolism, which may be evidence supported by response to heat stress with activation of intestinal anti-inflammation and immune system. These results suggest that when cows are exposed to a thermal environment, it can affect the interaction between the host intestine and microbes, which is closely related to the biological mechanism for maintaining intestinal integrity.

### Correlation between metabolite, DEGs, and microorganism

We integrated the transcriptome, metabolome, and intestinal microbial data and built a network composed of correlations. Through this, we were able to identify 34 positive correlations and 21 negative correlations in the control group and found 26 positive correlations and 47 negative correlations in the high-temperature stress experimental group. In particular, hexadecanoic acid and fructose, which are important indicators in the metabolome analysis results, were negatively correlated with eight DEGs in the control group and 15 DEGs in the high-temperature stress group, whereas myo-inositol was positively correlated with 11 DEGs in the high-temperature stress group. *GSTO1* (glutathione S-transferase omega 1), *MS4A3* (membrane spanning 4-domains A3), *CAVIN1* (caveolae associated protein 1), *TCAM1* (testicular cell adhesion molecule 1), *HSPB8*, and *HOMER3* (homer scaffold protein 3) are involved in maintaining cellular energy metabolism homeostasis, and their correlation with fructose is considered very meaningful [107]. In addition, *CD163*, *DEFB1* (defensin beta 1), *MPV17L* (MPV17 mitochondrial inner membrane protein like), and *TOMM7* (translocase of outer mitochondrial membrane 7) of Linoleic acid are involved in the regulation of proinflammatory cytokines, bacteria-induced antimicrobial peptides, and antioxidant-related genes [109]. We found that these genes were highly correlated with linoleic acid, which was related to their actions in the metabolome [71, 110]. In this context, *ATP6AP1L* (ATPase H^+^ transporting accessory protein 1 like), *GIPR* (gastric inhibitory polypeptide receptor), *KCNN1* (potassium calcium-activated channel subfamily N member 1), *KIR2DS1* (killer cell immunoglobulin-like receptor, two domains, short cytoplasmic tail 1), *NRN1* (neuritin 1), *PTGR1* (prostaglandin reductase 1) and *TGM1* (transglutaminase 1) are known to be involved in the oxidative stress response, mitochondrial metabolism, cell defense, and insulin secretion, and we found that these genes are related to linoleic acid. It was found that this was [71, 111–115]. In contrast, myo-inositol and six DEGs which *ATP6*, *HSPE1*, *IFI27* (putative ISG12(a) protein), *LCN2*, *LOC783945* (T cell receptor beta variable 5–5) and *SYT8* (synaptotagmin 8) showed positive correlations and were related to mitochondrial mechanisms, immune system, and insulin secretion [71, 116–118]. Metabolites and microorganisms showed significant correlations of 14 negative and three positive correlations, respectively, in the high-temperature stress test group. Among them, lactic acid and ornithine were negatively associated with *Oscillibacter*, *Parapedobacter*, and *Vicingus*, and these microorganisms are presumed to be associated with the process of accelerating the decrease in the amount of metabolites with glucose depletion in the body in a chronic heat stress environment [9, 45, 119]. In addition, *Oscillibacter* and *Parapedobacter* are known to maintain intestinal integrity by inducing the expression of antioxidant genes and preventing the accumulation of ROS or Malondialdehyde in damaged tissues, but the physiological characteristics of *Vicingus* in the animal intestine are not yet fully understood [45, 120, 121]. Linoleic acid shows significant associations with *Colidextribacter*, *Intestinimonas*, and *Pseudoflavonifractor*, which contribute to anti-inflammatory effects, oxidative stress, and butyrate production [122–124]. In this study, we explored the correlation between the blood metabolome, transcriptome, and gut microbiota and found that heat stress could affect the inflammatory response, redox, and cellular energy metabolism. However, we observed only a simple correlation and further research is required to understand the detailed mechanism.

## Conclusion

We conducted to analyze the differences of blood metabolites, transcripts, and intestinal microbes related to the stress response under heat stress conditions in dairy cows. In the high-temperature stress experimental group, metabolites including fructose and linoleic acid were significantly reduced, upregulated DEGs were shown pathway enrichments related to cellular energy metabolism and immunity system, and statistically significant microorganisms were related to mitochondrial activity and inflammatory response. Through multi-omics analysis, we confirmed the ability of heat stress to maintain homeostasis by altering the cellular energy mechanism, immune system, and microbial composition. Such results have been reported in several studies, especially alterations in immune response and cellular energy metabolisms. Considering the evidence for modulations of metabolome, transcriptome and microbiome levels by heat stress condition, more studies are needed for these interactions including direct phenotypic data and more large samples. In conclusion, using omics data, we found that the heat stress response induces oxidative stress, which induces an inflammatory response and that individuals maintain homeostasis through cellular energy metabolism and the immune system response. Thus, heat stress condition affects processes for energy production, immune response in metabolome, transcriptome and microbiome levels, and their correlation is also associated with inflammatory and antioxidant response, which can strongly support previous evidences. We believe that our research provides information that will help maintain dairy cow productivity in response to climate change.

## Methods

### Animal experiments

Animal experiments were conducted under the supervision of the Konkuk University IACUC Committee and in accordance with international guidelines (approval number KU20101). Eight Holstein cows were divided into a control group and an experimental group (with high-temperature stress), and the temperature and humidity of the chamber and water and feed intake were arbitrarily controlled during the 14-day experimental period. In the animal experiment, all animals were acclimatized for 7 days in a chamber environment of 21–22 °C and 50–60% humidity to remove other stresses. Afterwards, for 7 days, the control group was maintained at 25–26 °C and 35–50% humidity (THI 73–74), according to the temperature-humidity index (THI). The experimental group maintained 31–32 °C and 80–95% humidity (THI 85–87), and animals were exposed to heat stress condition from 9:00 am to 19:00 pm/day. After 14 days of experimentation, blood was collected from the tail veins of four animals in the control group and four in experimental group for RNA isolation and stored in Tempus Blood RNA tubes (Life Technologies, Carlsbad, CA, USA). To analyze the intestinal microbiome, feces were sampled from each individual at 7-days, and microbial DNA was extracted and stored (Noble Bio, Hwaseong, Gyeonggi-do, Korea).

### Metabolite analysis

For metabolite analysis, blood samples were extracted from 200 μL for each individual, and the detailed experimental steps were as follows. First, 1 ml of MeOH was added to the blood sample and mixed for 10 min using a mixer. And after storing for 1 h at -20 degrees for filtering the supernatant, centrifugation was performed at 10 min, 15000 rpm, and 4 °C., followed by concentration and drying using a vacuum concentrator. The sample dried by the concentration process was redissolved in MeOH, and 100 μL of the redissolved sample was placed in an e-tube and dried using a vacuum concentrator. The dried sample was reacted at 30 degrees for 90 minutes with 50 μL methoxyamine hydrochloride (20 mg/mL in pyridine) for oximation, and after the reaction was finished, it was filtered and put into a sampler vial, and GC-TOF-MS analysis was performed. For data analysis, raw data were converted to CDF format using Chroma TOF software, GC-TOF-MS data were digitized using the Metalign program, and processes such as peak selection, alignment, and baseline correction were performed. Finally, significant metabolites were identified, and their concentrations were plotted in the two groups using STAMP v2.1.3 [125].

### Bioinformatics analysis

#### RNA isolation and sequencing

RNA was isolated from blood samples according to the manufacturer’s instructions. For RNA purity evaluation, the Agilent 2100 Bioanalyzer and RNA Nano 6000 Assay Kit (Agilent Technologies, Santa Clara, CA, USA) were used, and only RNAs with an RNA integrity number >8 were used to construct a random cDNA library. For this purpose, we used the TruSeq Stranded Total RNA LT Sample Prep Kit (Illumina, San Diego, CA, USA) following the manufacturer’s instructions. On the Illumina platform, cDNA libraries generated BCL image files, and paired-end FASTQ raw read data were produced using the bcl2fastq Illumina package.

#### Mapping, counting and batch correction

We used FastQC v0.11.5, to check the sequence read quality (bioinformatics.babraham.ac.uk/projects/fastqc). We used HISAT2 v2.2.1 [126]. Index files were then created from the bovine reference genome (ARS-UCD 1.2.105), and the generated reads were mapped to the reference genome. Read-count data were created using the FeatureCounts program of the Subread package v2.0.1 [127]. In R, we used the RUVr function (k = 3 parameters) of the RUVSeq v1.24.0 package to perform batch correction [128]. In addition, we confirmed the correction effect by performing PCA on the data before and after batch correction using the ggfortify v0.4.11 package [129]. Next, we compared the gene expression levels between groups based on the corrected ret count data using the edgeR v3.32.0 package, found statistically significant DEGs using the generalized linear models method and tagwise dispersion for multiple factors following recommendations in the user’s manual [130].

#### Identification of DEGs and enrichment analysis

We compared the expression levels of the control group and the experimental group, and in the R program, DEGs were divided into upregulated and downregulated genes and were visualized by a volcano plot using the ggplot2 v3.3.3 package [131]. In addition, a heat map clustering was created using the pheatmap v1.0.12 package [132]. We performed functional analysis using DAVID for the DEGs and used GO and KEGG among the various databases in DAVID [133]. Additional functional analysis was performed by constructing gene networks for the up- and downregulated DEGs using ClueGO v2.5.10 (plug-in of Cytoscape v3.9.1) [134, 135].

#### Isolation of gut microbial DNA and Sequencing for 16s rRNA

Gut microbial DNA was isolated from the stool samples according to the manufacturer’s instructions. We created a DNA fragmentation-based random library according to the standard instructions of the 16S Metagenomic Sequencing Library preparation protocol (Illumina, San Diego, CA, USA). PCR amplification of the bacterial V3-V4 region was performed, and universal bacterial 16S rRNA primers were as follows: 5’-CCTACGGGNGGCWGCAG-3’ (341F) and 5’-GACTACHVGGGTATCTAATCC-3’ (805R). After purification of the PCR products according to a previously described protocol, adapters and index sequences were attached to the second PCR using the Nextera XT Index Kit (Illumina, San Diego, CA, USA), and the PCR products were purified for sequencing. The prepared libraries were sequenced on the Illumina MiSeq platform, and the generated BCL image files were created during the sequencing process for the gut microbial 16S rRNA V3-V4 region, and paired-end FASTQ raw read data were produced using the bcl2fastq Illumina package.

#### Gut microbiome analysis

First, raw read sequences were controlled by removing adapter sequences and correcting sequencing errors in the overlapping regions of reads using the fastp program [136]. Next, <400bp or >500bp reads using FLASH v1.2.11 [137]. CD-HIT-OTU removed low-quality sequencing errors and ambiguous and chimeric reads, and OTUs were formed by clustering sequences with a similarity of 97% or more [138]. Representative OTU sequences were assigned to the NCBI 16s rRNA database v2020.06.19, using BLAST+ v2.9.0 [139]. Subsequently, we checked the sequencing depth and estimated the alpha diversity (chao1, observed OTUs, and PD whole tree) using QIIME v1.9 [140]. In addition, to estimate the beta diversity, unweighted UniFrac and weighted UniFrac distances were calculated. The assigned OTUs were expressed as frequencies and relative abundances at the phylum and genus levels and used for microbial community comparison and function prediction between the two groups. Based on the OTU data, ggplot2 and randomcoloR v1.1.0.1 (R package) were used to plot the relative abundance and distance between the two groups in bar and principal coordinate analysis (PCoA) plots.

For microbial community comparison, the OTU table was normalized using the default parameters in MicrobiomeAnalyst [141]. We then performed LEfSe analysis, comparing differences between the two populations at the genus level [142]. We also used PICRUSt2 v2.4.1, to predict significant microbial functions, and the generated unstratified abundances based on the KEGG KO database were converted to the KEGG pathway database [143]. Finally, we compared difference between two groups in unstratified abundances through LEfSe.

### Data integration

We combined the results of the metabolome, transcriptome, and gut microbiome analyses, showing significant differences between the two groups (control and high-temperature stress experimental groups). In this process, Psycho v2.2.9 (R package) was used to calculate the relationship information of Metabolite-Microorganism and Metabolite-DEGs. Each correlation network was constructed using the Cytoscape software.

### Statistical analyses

The metabolite data was performed statistical analysis through multivariate statistical analyses (PCA, PLS-DA, and VIP) using the Simca-p+ program. And, genes with FDR (False Discovery Rate) <0.05 and |log_2_ FC (Fold Change| ≥ 1.0) were considered as DEGs. In functional analysis, DAVID and ClueGO were performed statistical analysis by EASE < 0.1, *p* < 0.05 (DAVID) and two-sided tests based on hypergeometry with Bonferroni step down (*p* < 0.05, ClueGO). In LEfSe analysis, LDA effect size ± ≥2 was used for comparing differences between two groups. Finally, correlation network between datasets was considered the statistical criteria using Spearman’s method in Psycho v2.2.9 with *p* < 0.05 and *r*^2^ ± ≥ 0.95 [144].

## Supporting information

S1 Table The identified metabolites. (XLSX)

S2 Table Detailed raw reads information in mRNA. (XLSX)

S3 Table The identified DEGs. (XLSX)

S4 Table Results of functional analysis with DAVID. (XLSX)

S5 Table Results of functional analysis with ClueGO. (XLSX)

S6 Table Detailed raw reads information in 16S rRNA. (XLSX)

S7 Table OTU abundance. (XLSX)

S8 Table Differentially abundant microbes using LEfSe with filtered counts. (XLSX)

S9 Table Results of gene prediction with PiCRUST2. (XLSX)

S10 Table Correlation network. (XLSX)
